# Retrospective investigation of the origin and epidemiology of the dengue outbreak in Yunnan, China from 2017 to 2018

**DOI:** 10.3389/fvets.2023.1137392

**Published:** 2023-04-03

**Authors:** Liang Cao, Ziping Yu, Haiqiang He, Xiaofang Guo, Chun Wei, Xuancheng Zhang, Junduo Bao, Chenghui Li, Hongning Zhou, Jialiang Xin, Fulong Nan

**Affiliations:** ^1^College of Laboratory, Jilin Medical University, Jilin, China; ^2^Institute of Virology, Wenzhou University, Wenzhou, China; ^3^College of Veterinary Medicine, Jilin University, Changchun, China; ^4^Yunnan Institute of Parasitic Diseases, Pu'er, Yunnan, China; ^5^Collage of Agriculture, Yanbian University, Yanbian, China; ^6^College of Veterinary Medicine, Sichuan Agricultural University, Chengdu, China; ^7^Department of Special Medicine, School of Basic Medicine, Qingdao University, Qingdao, China

**Keywords:** dengue virus, epidemiology, phylogenetic analysis, Yunnan, 2017–2018

## Abstract

Since 2013, a dengue epidemic has broken out in Yunnan, China and neighboring countries. However, after the COVID-19 pandemic in 2019, the number of dengue cases decreased significantly. In this retrospective study, epidemiological and genetic diversity characterizations of dengue viruses (DENV) isolated in Yunnan between 2017 and 2018 were performed. The results showed that the dengue outbreak in Yunnan from 2017 to 2018 was mainly caused by DENV1 (genotype I and genotype V) and DENV2 (Asia I, Asia II, and Cosmopolitan). Furthermore, correlation analysis indicated a significant positive correlation between the number of imported and local cases (correlation coefficient = 0.936). Multiple sequence alignment and phylogenetic divergence analysis revealed that the local isolates are closely related to the isolates from Myanmar and Laos. Interestingly, recombination analysis found that the DENV1 and DENV2 isolates in this study had widespread intra-serotype recombination. Taken together, the results of the epidemiological investigation imply that the dengue outbreak in Yunnan was primarily due to imported cases. This study provides a new reference for further investigations on the prevalence and molecular epidemiology of DENV in Yunnan, China.

## 1. Introduction

The dengue virus (DENV) is a highly prevalent mosquito-borne pathogen, which enters the human body through the bite of *Aedes aegypti* and *Aedes albopictus* mosquitoes (1). Clinically, DENV leads to dengue fever (DF), dengue hemorrhagic fever (DHF), and dengue shock syndrome (DSS), causing a serious threat to human life and health (2). The DENV belongs to the *Flaviviruses* in the family *Flaviviridae*. It is an enveloped, single-stranded, negative-sense, RNA virus with a genome (~11.0 kb) containing only one open reading frame (ORF), which encodes for three structural proteins and seven non-structural proteins. In general, DENVs can be grouped into four different serotypes (DENV 1–4) based on the antigenic differences of the E protein, and its serotypes can be further divided into different genotypes. It is worth noting that there is no cross-protection between different serotypes of DENV (3, 4).

The E protein (~495 amino acids, AA) is the most fully exposed protein on the surface of mature infectious virus particles and contains crucial immunological epitopes associated with virus neutralization. Moreover, the E protein is the main virulence factor that plays an important role in mediating the adsorption of viruses in host cells, membrane fusion, virus assembly, and virus maturation (5). Three domains (EDI, EDII, and EDIII) are formed in the spatial structure based on the determined crystal structure of the E protein. Among these, EDII, which is located at the amino terminus of the E protein, contains a fusion peptide structure that mediates the fusion of the virus and the target cell (fusion loop, 98–110 AA) (6). Meanwhile, EDIII facilitates the adsorption of viruses to target cells and is considered to contain the receptor binding region as well as the most important neutralizing epitope (7–9).

DENVs have been popular in more than 100 countries, especially in tropical and subtropical regions. About half of the world's population lives in dengue-affected areas, and more than 50 million people get infected every year (10). The global DF epidemic is becoming more and more serious, threatening global public health (11). In recent years, dengue epidemics have broken out in several regions of China, such as in Guangdong, Zhejiang, Yunnan, Henan, and Hunan (12–15). In particular, the Yunnan Province of China has been reported as endemic for dengue with numerous cases reported annually. In 2008, a dengue epidemic caused by DENV1 occurred in Ruili City, Yunnan Province, China, a region that is adjacent to Myanmar. In 2013 and 2015, large-scale dengue outbreaks occurred in Xishuangbanna, Yunnan Province, China, which is also adjacent to Myanmar and Laos (15, 16). From 2015 to 2016, Dengue outbreaks continue to occur in the border areas between China, Laos, Myanmar, and Vietnam (17). Interestingly, after the COVID-19 pandemic in 2019, the number of dengue cases has dropped significantly. However, no relevant reports are available on the dengue prevalence in Yunnan Province from 2017 to 2018, which is crucial to the traceability and epidemiological detection of the dengue epidemic. To explore the relationship between the COVID-19 outbreak and the substantial reduction of dengue cases, it is necessary to study the epidemic trend and characteristics of dengue from 2017 to 2018, before the COVID-19 outbreak. In this report, we investigated the prevalence of DENV in Yunnan Province, China from 2017 to 2018. This study could fill the gap in the epidemiological surveillance of DENV from 2017 to 2018. Moreover, improving the comprehension of the molecular characteristics and evolutionary trends of the circulating DENV strains.

## 2. Materials and methods

### 2.1. Ethical considerations

This study was approved by the Yunnan Institute of Parasitic Diseases and Jilin Medical University (No. 2020_GKJJ028). Written informed consent was obtained from all patients and/or legal guardians of the children involved in this study.

### 2.2. Sample collection

A total of 172 human serum samples, which were positive for DENV NS1 and negative for DENV IgG, were obtained from the Yunnan Institute of Parasitic Diseases. The samples were stored at −80°C before further analyses.

### 2.3. RNA extraction and real-time polymerase chain reaction (RT-PCR)

Total viral RNA was extracted from human serum samples using the FastPure Cell/Tissue Total RNA Isolation Kit V2 (Vazyme Biotech Co., Nanjing) following the manufacturer's protocol. The primer pairs used were designed based on the DENV reference sequence from NCBI (Supplementary Tables 1–3). The HiScript^®^ II One-Step RT-PCR Kit (Dye Plus) was used for PCR amplification (Vazyme Biotech Co., Nanjing). The 5 mins TA/Blunt-Zero Cloning Kit (Vazyme Biotech Co., Nanjing) was used to construct the recombinant vectors, which were cloned in *Escherichia coli* (*E. coli*, DH5α) before sequencing.

### 2.4. Data sources and statistical analysis

The dengue reference sequences used in this study were obtained from the NCBI database and counted. The reported cases in Vietnam, Laos, Cambodia, Philippines, Singapore, and Myanmar were collated from the World Health Organization (WHO) Regional Office for the Western Pacific's Institutional Repository for Information Sharing (WPRO IRIS, http://apps.who.int/iris/). The reported cases in Yunnan Province of China were obtained from the National Health Commission of the People's Republic of China (http://www.nhc.gov.cn/). The data were analyzed using the SPSS software.

### 2.5. Phylogenetic analysis

The DENV genome sequences and the E gene obtained in this study have been deposited in GenBank under the accession numbers OQ652963—OQ652975. Reference genome sequences for DENV were obtained from NCBI. The sequences were assembled using the SeqMan software (DNASTAR Inc., Madison, Wisconsin, USA) and were aligned using MegAlign (DNASTAR Inc., Madison, Wisconsin, USA), with the Clustal W alignment method for genomic and AA similarity analyses. Phylogenetic trees were constructed using the neighbor-joining (NJ) method and a p-distance model (bootstrap analysis with 1,000 replicates).

### 2.6. Recombination analysis

Recombination event analysis was carried out by analyzing the E gene sequences of DENV1 and DENV2. The RDP4 (http://web.cbio.uct.ac.za/~darren/rdp.html) software (RDP, Bootscan, MaxChi, GENECONV, Chimera, SiScan, and 3Seq) was used for preliminary screening of recombinant strains, while the SimPlot software (http://sray.med.som.jhmi.edu/RaySoft) was used to analyze the breakpoints and the sequences of recombination and parental lineages.

## 3. Results

### 3.1. Epidemic trend of dengue in Yunnan Province of China and neighboring countries

**Figure 1:**
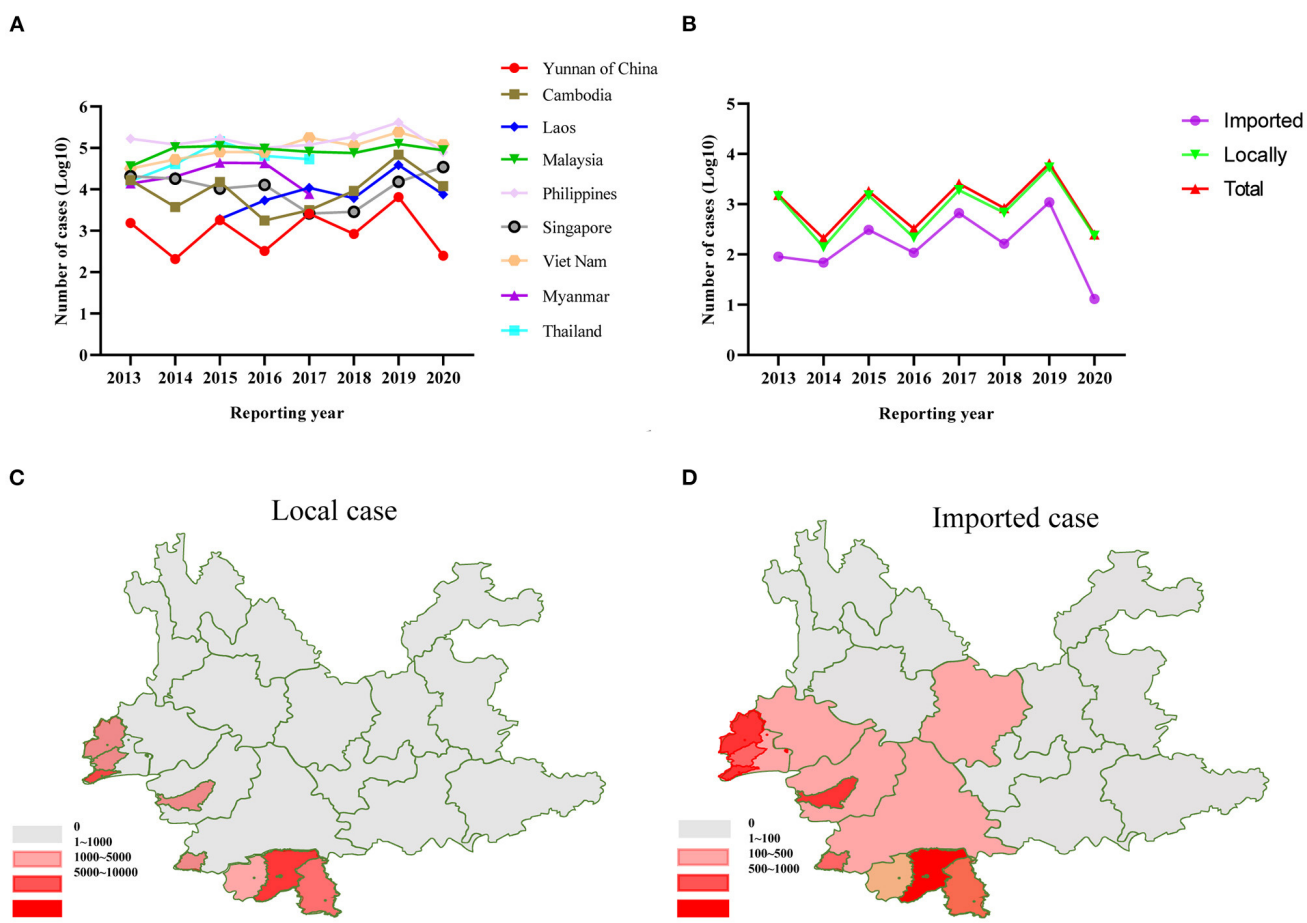
**(A)** The number of dengue cases in Yunnan Province of China and neighboring countries from 2013 to 2020. **(B)** The number of local cases and imported cases in Yunnan Province of China from 2013 to 2020. **(C)** Regional distribution map of local cases from 2013 to 2020 in Yunnan, China. **(D)** Regional distribution map of imported cases from 2013 to 2020 in Yunnan, China.

**Figure 2:**
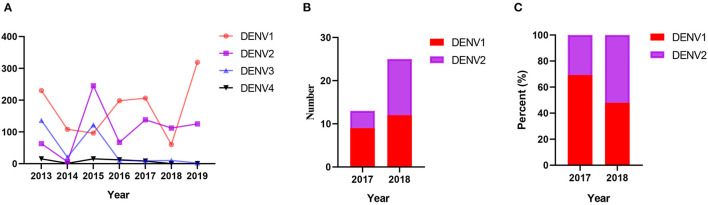
**(A)** The number of DENV1-4 strains in the GenBank datasets from 2013 to 2019 in Yunnan, China. **(B)** The number of different DENV strains in this study. **(C)** The percentage of different DENV strains in this study.

According to WHO, the number of dengue cases in Yunnan Province of China and neighboring countries remained at a high level from 2013 to 2019. After the COVID-19 outbreak in 2019, the number of dengue cases in neighboring countries in 2020 has not decreased significantly, but the number of dengue cases in China has dramatically decreased (Figures 1A, B). A comparative analysis of the distribution areas of local and imported cases revealed that the main outbreak areas overlapped, mainly in Xishuangbanna and Ruili in Yunnan, China (Figures 1C, D). Additionally, the correlation analysis between the number of imported and the local cases indicated a significant positive correlation (correlation coefficient = 0.936). However, since there are no detailed publicly available data, it is impossible to determine the number of DENV1-4 isolates among the dengue cases from 2013 to 2019. Therefore, this study determined the number of DENV1-4 isolates in Yunnan from 2013 to 2019 using the GenBank data and found that the change in the trend of DENV1-4 strains is consistent with the trend of the dengue epidemic. However, genome sequencing data for DENV in Yunnan Province. China after 2019 are inadequate in the GenBank database (Figure 2A).

### 3.2. Screening for DENV prevalence in clinical samples

In this study, among the 172 human serum samples collected, 38 (22.1%) serum samples were positive for DENV. From this, 13 of 72 (18.1%, 9 DENV1, 4 DENV2) serum samples were positive in 2017, and 25 of 100 (25.0%, 12 DENV1, 13 DENV2) serum samples were positive in 2018. These show that the proportion of DENV2 strains detected rose from 30.8 to 52.0% from 2017 to 2018 (Figure 2). These results are consistent with the statistical data of DENV in GenBank. In the genome sequencing data, the repeated sequences were removed to obtain three complete sequences (OQ652963–OQ652965) and ten E gene sequences (OQ652966–OQ652975). Among these, the YN/324 and YN/017 isolates were from Myanmar travelers, and the YN/117 isolate was from a Laos traveler.

### 3.3. Sequence analysis

The genome and the E gene sequences of the strains used in this study were obtained by overlapping polymerase chain reaction. Among these sequences, the YN/117 isolate was obtained from a Laos traveler, while the YN/017 and YN/324 isolates were obtained from Myanmar travelers. The other strains analyzed in this study were obtained from local residents. The genome and the ORF of the YN/RL isolate include 10,735 nucleotides (nts) and 10,179 nts (3,393 AA), including 94 and 465 nts in the 5′ and 3′ UTRs, respectively. The YN/RL isolate lacks one nt (79 nt) in the 5′ UTR, but the secondary RNA structure of the 5′ UTR did not change significantly. Interestingly, the genomes of the YN/MH strain and the YN/017 isolate have different lengths. The ORF and 5′ UTR were 10,176 nts (3,391 AA) and 96 nt, in length, respectively. The difference is that compared with the YN/MH strain and DENV2-SS (New Guinea C) isolate, the YN/017 isolate lacks 13 nts (10,270–10,282 nt) in the 3′ UTR, resulting in significant changes in the secondary RNA structure (Supplementary Figures 1, 2). Furthermore, the E gene of these sequences all contain 1,485 nts and encode 495 AA.

### 3.4. Multiple sequence alignment and phylogenetic analysis

**Figure 3:**
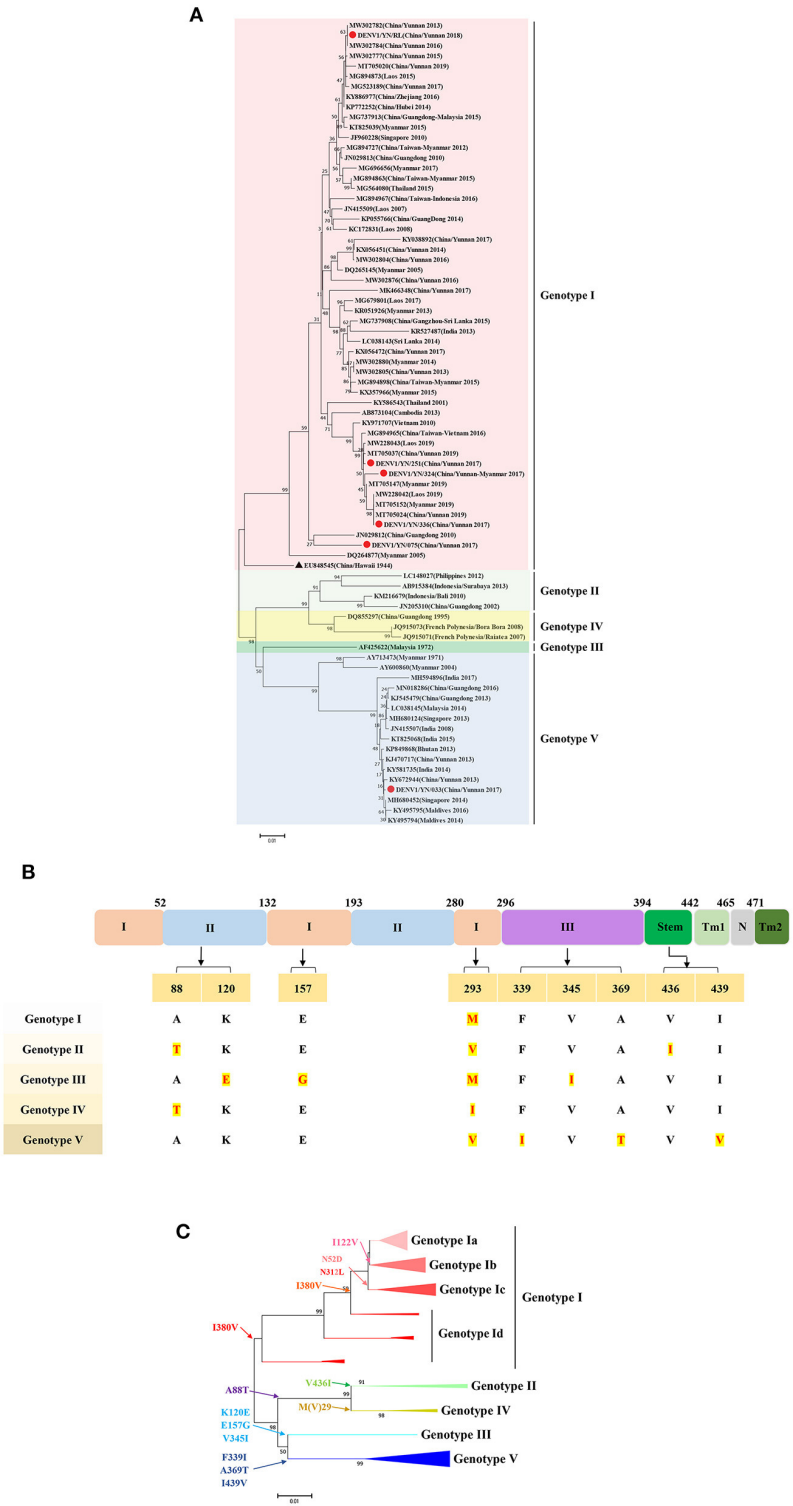
**(A)** Phylogenetic tree of DENV-1 epidemic strains in Yunnan, China. The maximum likelihood (ML) trees were built using 1,000 bootstrap replicates. The red circles indicate the strains detected in this study. **(B)** The characteristics of AA variations among different genotypes of DENV2. **(C)** Relationship between the evolutionary tree branching and AA mutations in genotype I of DENV-1.

To perform the genetic evolution analysis, the reference sequences of the classic DENV strains from various countries or regions, with a focus on the three countries adjacent to Yunnan Province of China: Myanmar, Laos, and Vietnam, were obtained from NCBI. Intriguingly, the phylogenetic divergence analysis based on the E gene sequences of DENV1 revealed that the YN/033 strain belongs to genotype V, which was first detected in Yunnan Province of China after 2013. The other DENV1 isolates in this study, namely YN/251, YN/324, YN/336, YN/075, and YN/RL, were clustered in a branch representing genotype I. The YN/251 and YN/336 isolates were distributed in one cluster of the ML tree, with the closest relationships to the YN/324 isolate (Yunnan/Vietnam traveler), Vietnam isolate (2010, KY971707), and Thailand isolate (2001, KY586543). It is worth noting that these isolates were continuously detected in the Yunnan Province of China, as well as in neighboring countries (Laos, Myanmar, and Vietnam) from 2013 to 2019. These results indicate that these isolates have become superior strains and have spread stably in the local area. The YN/RL strain was in another cluster, with the closest relationships to the Laos isolate (2015, MG894873) and the China/Zhejiang isolate (2016, KY886977). Similarly, the YN/RL isolate was continuously detected in Yunnan Province of China, as well as other provinces (Zhejiang, Guangdong, and Hubei) and neighboring countries (Laos, Myanmar, and Singapore) from 2013 to 2019. Meanwhile, the YN/075 isolate is genetically distant from the other isolates in this study and has a close relationship with the Guangdong (2010, JN029812) and USA/Hawaii isolates (1944, EU848545, DENV1-SS) (Figure 3A).

Further analysis of the relationship between the evolutionary tree branching and AA substitution of DENV1 revealed that AA variations at 10 positions determine the evolutionary differences of the genetic subtypes. More specifically, V(I)293M results in genotype I, V436I results in genotype II, K120E, E157G, V345I result in genotype III, M(V)293I results in genotype IV, and F339I, A369T, and I439V result in genotype V. The DENV1 genotype 1 can be divided into 4 subgroups based on the AA variation: I122V-genotype Ib, N52D and V312L-genotype Ic, and I380V-genotype Id (Figures 3B, C).

**Figure 4:**
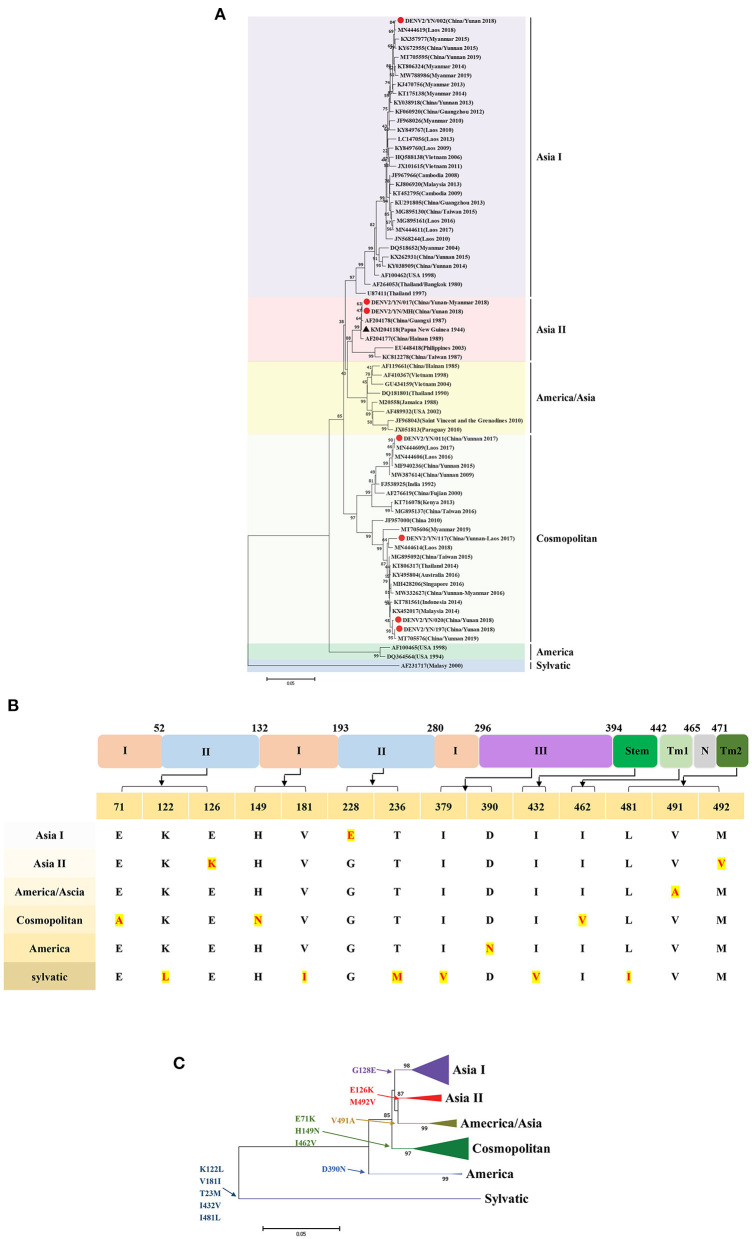
**(A)** Phylogenetic tree of DENV-2 epidemic strains in Yunnan, China. The ML trees were built using 1,000 bootstrap replicates. The red circles indicate the strains detected in this study. **(B)** The characteristics of AA variations among different genotypes of DENV2. **(C)** Relationship between evolutionary tree branching and AA mutations in genotype I of DENV-2.

Similarly, the phylogenetic analyses revealed that the strains in this study belong to Asia I (YN/002 isolate), Asia II (YN/017 and YN/MH isolates), and Cosmopolitan (YN/011, YN/117, YN/020, and YN/197 isolates) types. The YN/020 and YN/197 isolates have close relationships with the YN/117 isolate (Yunnan/Laos traveler), Laos isolate (2018, MN44614), and Malaysia isolate (2014, KX452017). Compared with the YN/117 isolate, YN/020, and YN/197 isolates, the YN/011 isolate was in another cluster of Cosmopolitan type, with the closest relationship to the India isolate (1992, FJ538925). It is worth noting that the Cosmopolitan type isolates in this study have been detected in Yunnan Province of China (including foreign tourists) and multiple countries (Australia, Singapore, Myanmar, Thailand, Laos, Indonesia, and Malaysia) from 2014 to 2019. The above results suggest that these strains may have been the superior strains during the global epidemic. The YN/MH isolate has close relationships with the YN/017 isolate (Yunnan/Myanmar traveler) and the Papua New Guinea strain (1944, KM204118, DENV2-SS). Meanwhile, the YN/002 strain is genetically distant from other isolates in this study and has a close relationship with the Laos isolate (2018, MN44619) (Figure 4A).

The combined results of multiple sequence alignment and evolutionary analysis revealed that 8 AA variations are related to genetic evolution. Specifically, D390N results in the America type, E71K, H149N, and I462V result in the Cosmopolitan type, V491A results in the America/Asia type, G128E results in Asia I type, and E126K and M492V result in the Asia II type (Figures 4B, C).

### 3.5. Homology and AA mutation analysis

**Figure 5:**
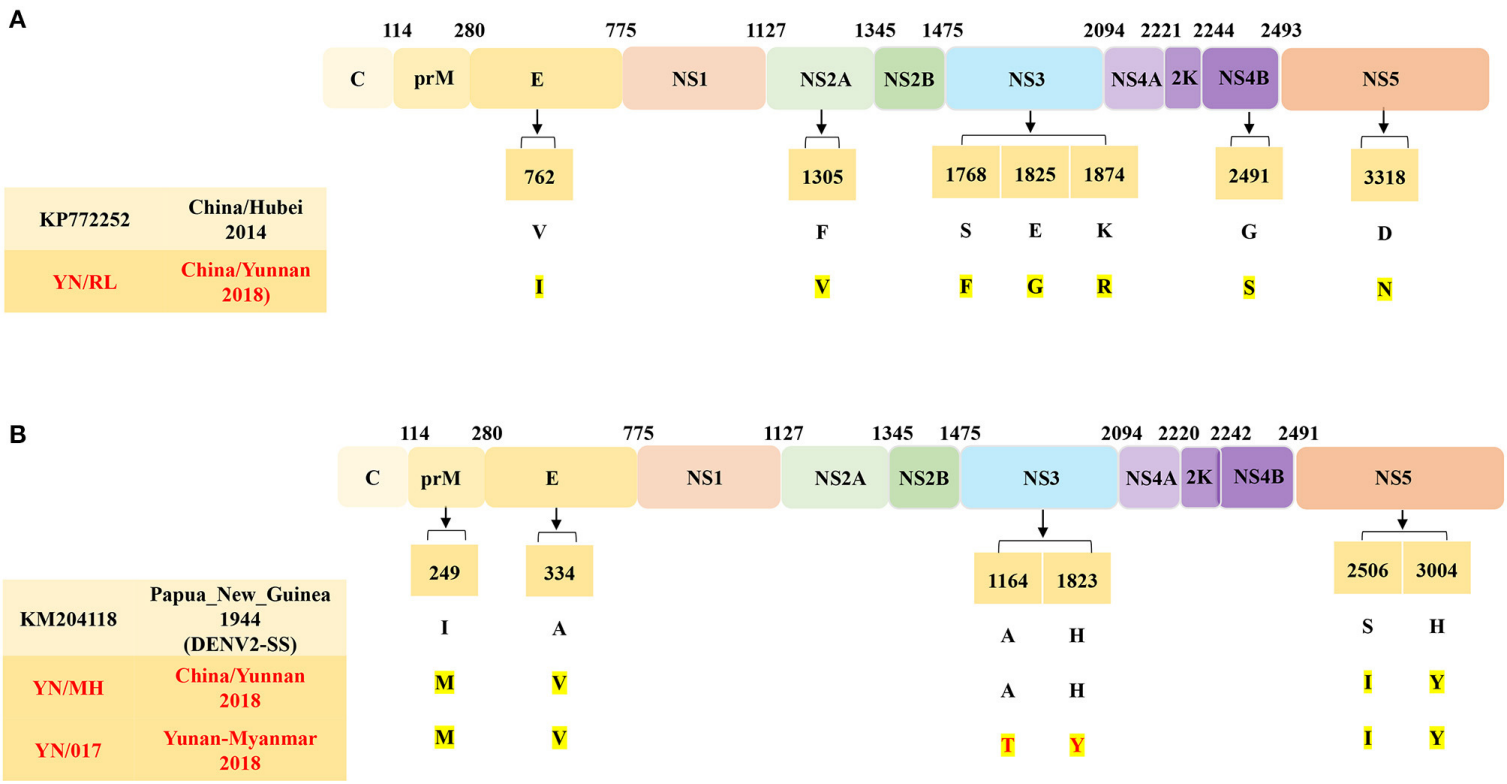
**(A)** AA mutations in the E gene of DENV1. **(B)** AA mutations in the E gene of DENV2.

A comparison of the complete ORF sequences revealed that the YN/RL isolate shares 90.5–97.2% identity with the DENV1 reference sequence. Based on nucleotide similarity analysis of the DENV1 E protein, the isolates in the study share 89.7–100.0% similarity with the DENV1 reference sequence (Supplementary Table 4). Compared to the DENV1-SS E protein, the DENV1 isolates in this study have 22 AA mutations, of which eight AA mutations have never been reported (Figure 5A). Specifically, the YN/324 (Yunnan/Myanmar traveler) isolate contains four AA mutations (H89Q, N92A, V91A, and C92G) that were present in the first ED II domain of the E protein. Moreover, compared to the closest China/Hubei strain (2011, KP772252), a total of 7 AA mutations occurred in the CDS of the YN/RL strain.

Similarly, the complete ORF of the YN/MH strain shared 91.9–93.9% nt identity with the reference sequence of DENV2. Multiple sequence alignment revealed that the E protein of DENV2 strains in this study has 89.2–99.9% identity to the reference sequence (Supplementary Table 5). Compared to the E protein of the Papua New Guinea strain (1944, KM204118, DENV2-SS), the DENV2 strains in this study have 17 AA mutations, of which three AA mutations have never been reported (Figure 5B). Compared to the closest DENV2-SS strain, a total of four AA mutations occurred in the CDS of the YN/MH strain and the YN/017 strain (Yunnan/Myanmar traveler). The difference is that compared with the DENV2-SS and the YN/MH strains, the YN/017 strain (Yunnan/Myanmar Traveler) has two AA mutations in the NS3 protein.

### 3.6. Recombination analysis

**Figure 6:**
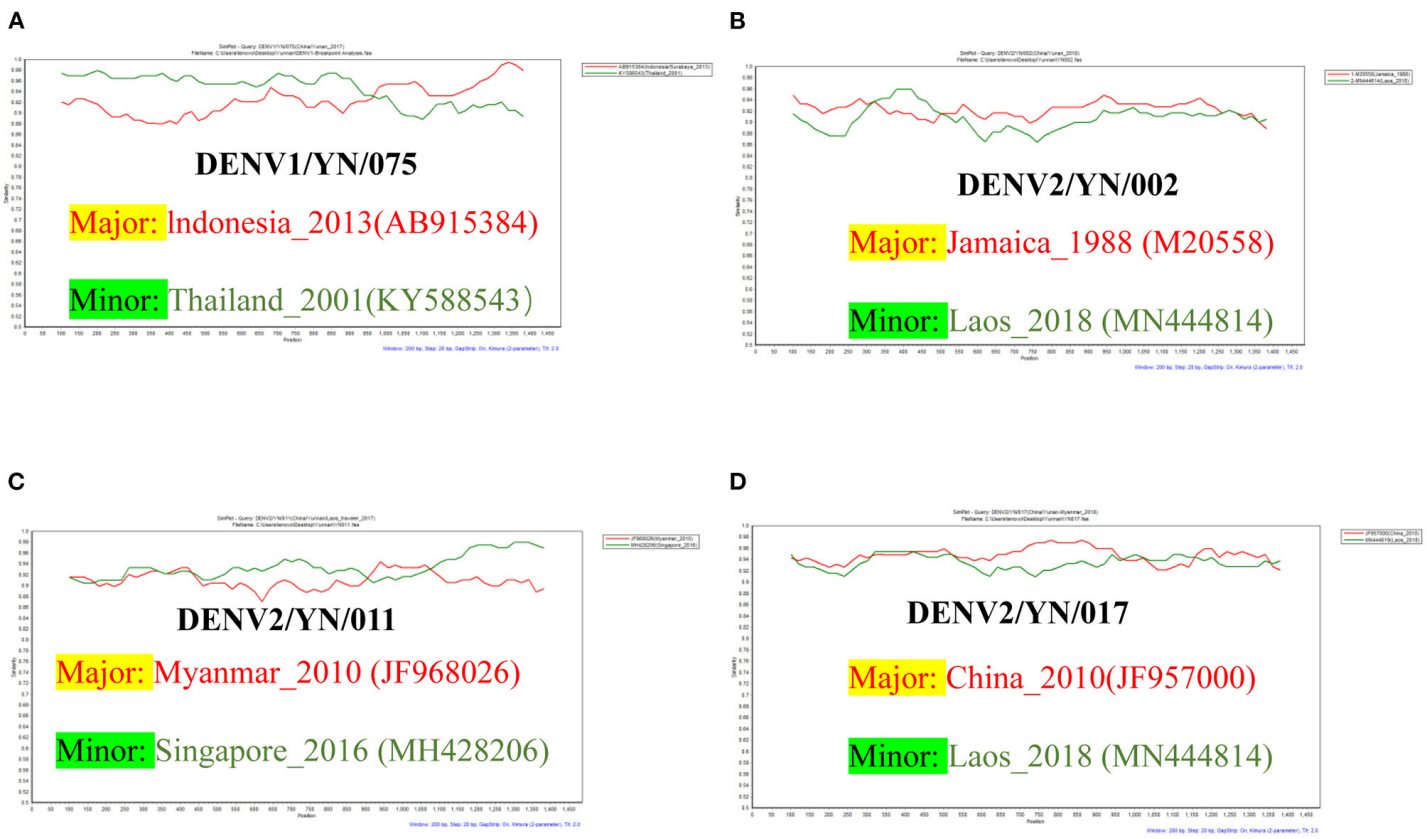
**(A)** Recombination analyses of the YN/075 isolate, which is composed of the parental strain (Indonesia/Surabaya, AB915384) and the minor strain (Thailand, KY586543). **(B)** Recombination analyses of the YN/002 isolate, which is composed of the parental strain (Jamaica, M20558) and the minor strain (Laos, MN444614). **(C)** Recombination analyses of the YN/011 isolate, which is composed of the parental strain (Myanmar, JF968026) and the minor strain (Singapore, MH428206). **(D)** Recombination analyses of the YN/017 isolate, which is composed of the parental strain (China, JF957000) and the minor strain (Laos, MN444619).

Multiple studies have confirmed that DENV has a widespread intra- and inter-serotype recombination. The combined results of the RDP4 and SimPlot analyses showed that four strains in this study existed *via* intra-serotype recombination. Among these, the genome sequence of the YN/075 strain has a single breakpoint, which is composed of the major strain (Indonesia/Surabaya strain, AB915384, 2013) and the minor strain (Thailand strain, KY586543, 2001). Interestingly, the genome sequence of the YN/002, YN/011, and YN/017 strains all contained multiple breakpoints. Moreover, no recombination between different serotypes has been detected (Figure 6).

## 4. Discussion

Yunnan Province of China has a subtropical and tropical monsoon climate with numerous flora and fauna. The southern part of Yunnan Province of China, such as the Xishuangbanna area, is located on the northern edge of the tropics with lush vegetation. The annual average temperature in the area is maintained between 18.9 and 22.6°C, which is very suitable for the growth and reproduction of mosquitoes. Moreover, Yunnan has close economic exchanges with Southeast Asian countries and is also a popular holiday and tourist destination. This has resulted in very high population mobility in the border areas of Yunnan, which facilitated the spread of mosquito-borne viruses such as DENV, Japanese encephalitis, chikungunya, and Zika (18, 19).

Since Liu et al. first reported the detection of DENV in Yunnan Province in 2006, large-scale dengue outbreaks have occurred in Yunnan every 2 years since 2013, involving different DENV serotypes (20). More specifically, DENV1 was the main serotype that caused dengue epidemics in 2013–2016, DENV2 was mainly involved in 2015, DENV3 was mainly concentrated in 2013 and 2015, while DENV4 was only scattered sporadically (21–23). Notably, the dengue outbreak areas, such as Jinghong, Mengla, Ruili, Jiangcheng, and Menglian, were mainly focused on border port cities with Myanmar, Vietnam, and Laos. In other words, Yunnan was a province that borders dengue-endemic Southeast Asian countries. In the past, it was generally believed that the dengue outbreak in Yunnan was caused by local cases spreading through mosquito bites or mosquitoes spreading across borders. In response to the COVID-19 pandemic, China implemented strict restrictions on cross-border travel to prevent disease spread. As a result, the cross-border floating population has decreased significantly. Surprisingly, the number of dengue cases also decreased from 6,840 in 2019 to 260 in 2020. On the contrary, the number of dengue cases in the Southeast Asian dengue-endemic countries adjacent to Yunnan did not drop significantly. Subsequently, a correlation analysis of the regional distribution and trends of local and imported cases revealed a significant positive correlation. The results indicated that the size of the cross-border floating population in Yunnan Province is related to the dengue outbreak. In a study that further explored the sources of the dengue outbreak in Yunnan of China, the results of homology comparison and genetic evolution analysis revealed that there was a high homology and close relationship between the local strains and the strains from Laos and Myanmar travelers. Based on these, it was speculated that the dengue outbreak in Yunnan was mainly caused by imported cases. Li et al. used the 2013–2020 epidemiological and viral genome data from Yunnan and neighboring countries to establish a multivariate statistical model for analysis (24). It was concluded that Yunnan is a regional sink for the DENV lineage movement and that the dengue incidence between 2013 and 2019 in Yunnan was closely linked with the international importation of cases. These findings are consistent with our results.

Interestingly, the positivity rate of DENV2 has significantly increased from 2017 to 2018 in Yunnan, China. Statistics show that before 2015, DENV1 was the main prevalent serotype in the world. However, since 2015, the scale and frequency of dengue epidemics caused by DENV2 in various countries and regions have been increasing. DENV2 has maintained a high infection rate in Yunnan since 2014. Moreover, in 2015 and 2018, the number of DENV2 dengue cases far exceeded other serotypes. These results suggest that DENV2 and DENV1 may alternately become the main epidemic serotypes in Yunnan Province, China. Genetic evolution analysis revealed that the DENV strains circulating in Yunnan were mainly genotype 1 of DENV1 and Cosmopolitan type of DENV2. It is particularly important to note that some strains have existed stably in Yunnan and neighboring countries for a long time. For example, the DENV1 isolates (YN/RL, YN/251, YN/324, and YN/326) and DENV2 isolates (YN/002, YN/011, YN/117, YN/197, and YN020) were repeatedly detected in Yunnan and neighboring countries from 2013 to 2019. On the contrary, genotype V isolates (such as YN/033) disappeared after 2013 in Yunnan and reappeared in 2017. Further studies are needed to trace the origin of these strains. Whether genotype V will become a new epidemic subtype requires attention and continuous follow-up investigation.

Mutation and recombination play decisive roles in the evolution of viruses, especially in RNA viruses. The 5′ and 3′-UTRs of viruses play important roles in the efficiency of viral RNA translation and genome replication. Song et al. confirmed that the 5'-UTRs of flaviviruses serve as internal ribosome entry sites (25). Meanwhile, Yan et al. used a virus-induced reporting gene system to show that the deletion of nts 10,663–10,677 and 10,709–10,723 facilitates viral translation (26). In the present study, compared with the DENV1-SS or DENV2-SS isolates, the YN/RL and YN/MH isolates were shown to have multiple base deletions or mutations at the 5' and 3'-UTRs. Specifically, the YN/017 isolate has 13 nts (10,270–10,282 nt) deletions in the 3′ UTR. These nt changes led to significant changes in the RNA secondary structure. Whether these changes relate to the level of replication and translation of the isolates remains to be confirmed by further research.

Herein, the prM protein could promote the maturation of the virus and increase its infectivity, as well as induce the production of protective antibodies. The E protein contains immunologically important epitopes associated with virus neutralization and has become a potential target protein for vaccine and neutralizing antibody therapy. Deng et al. identified a broad flavivirus cross-neutralizing monoclonal antibody that can recognize a new epitope in the E protein fusion loop (EDII, ^98^DRXW^101^) (27). While Thullier et al. indicated that the murine monoclonal antibody 4E11 neutralizes DENV of all serotypes by binding to the 296–400 segment of the major DENV envelope glycoprotein (DE) (28). Moreover, Chin et al. found that the E protein domain III of DENV could competitively inhibit virus entry (29). In the present research, multiple AA mutations were found on the E protein of these isolates. In particular, 12 AA mutations were unique to the isolates in this study and have never been reported. Furthermore, a single mutation (S139N) in the PrM protein of YN/ML and YN/017 isolates was found. Thus, AA substitution in both prM/E proteins may greatly affect the immunogenicity and receptor affinity of DENVs. It is worth noting that the E genes of these isolates have extensive intra-serotype recombination, which can accelerate the mutation and evolution of the virus.

In this study, these isolates also showed multiple AA mutations in non-structural proteins, mainly in NS3 and NS5. Non-structural proteins also act in virus replication and evasion of host immune responses. For instance, Ye et al. indicated that a single silent mutation G66A in the NS2A gene of JEV abolished the production of NS1 *in vitro* and reduced neurovirulence and neuroinvasiveness in mice (30). The NS3 is a multifunctional protein with helicase, RNA-stimulated nucleoside triphosphatase (NTPase), and RNA 5'-triphosphatase (RTPase) activities. Meanwhile, NS5 is responsible for the replication of the viral genome, RNA capping, and suppression of host interferon responses (31). Thus, AA substitutions in non-structural proteins may greatly affect the efficiency of viral replication.

From 2013 to 2020, Yunnan had many large-scale dengue outbreaks. However, multiple studies have been divergent on the source and prevalence of dengue in Yunnan. This study systematically analyzed dengue epidemic trends, geographical distribution, and genetic evolution in Yunnan and surrounding countries. Multiple pieces of evidence imply that the dengue outbreak in Yunnan was mainly caused by imported cases. Moreover, DENV2 and DENV1 may alternately become the main epidemic serotypes in Yunnan Province, China. Although the prevalence of dengue fever in China has shown a downward trend in recent years, there is still the possibility of repeated epidemics and outbreaks of dengue fever. These could seriously affect the public health system and cause serious economic losses. Therefore, the prevention, control, and monitoring of dengue should still be a priority. We hope that our findings could serve as a reference for future studies on the tracing, epidemic trend, and variation of DENV in Yunnan province, China.

## Data availability statement

The data presented in the study are deposited in the GenBank repository, accession numbers OQ652963—OQ652975.

## Ethics statement

Ethical review and approval was not required for the study on human participants in accordance with the local legislation and institutional requirements. Written informed consent from the patients/participants was not required to participate in this study in accordance with the national legislation and the institutional requirements.

## Author contributions

LC, ZY, HH, and XG performed the experiments, analyzed the data, and wrote the manuscript. CW, XZ, and JB helped in writing the manuscript. CL, HZ, JX, and FN participated in sample collection, research testing, and research design. All authors read and approved the final version of the manuscript.
