# Supplementary figures and images for: Retrospective investigation of the origin and epidemiology of the dengue outbreak in Yunnan, China from 2017 to 2018
Source: Front Vet Sci. 2023 Apr 3;10:1137392. doi: 10.3389/fvets.2023.1137392 (PMC10132138; doi:10.3389/fvets.2023.1137392)

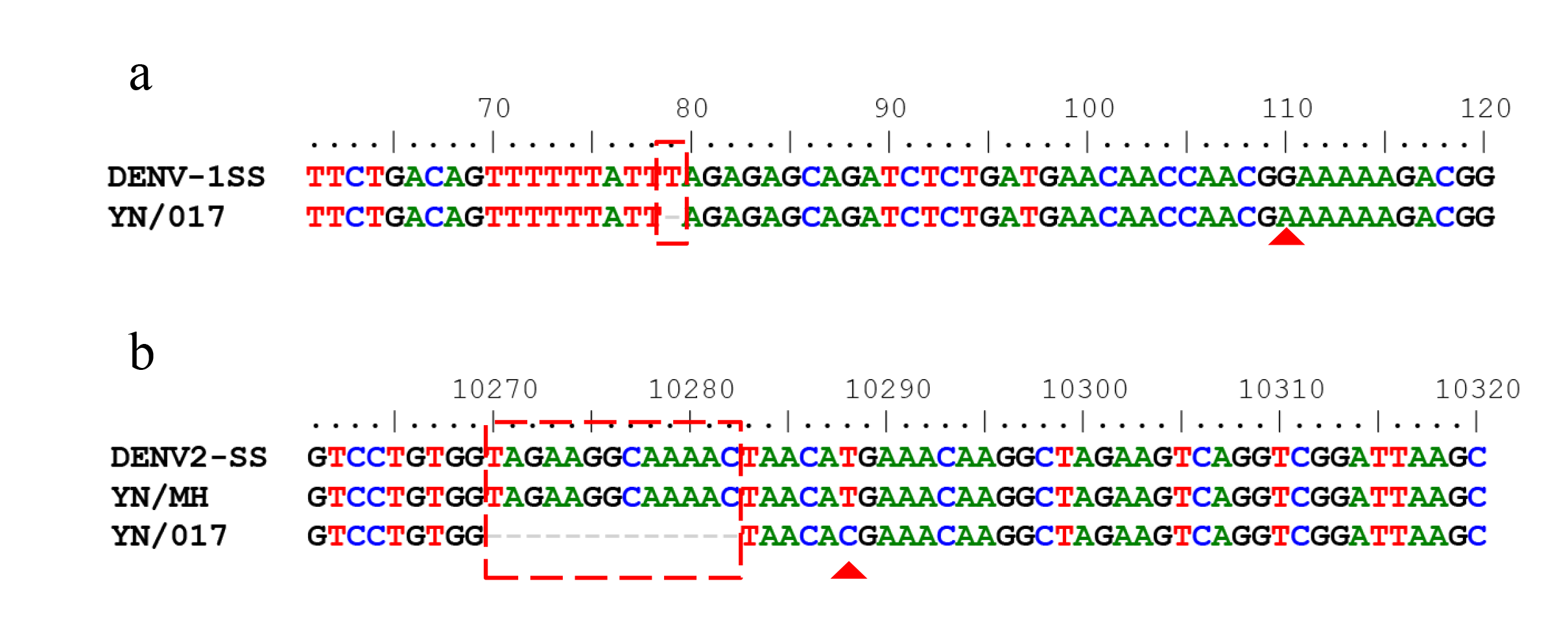

Supplement: Supplementary file 1 [file Image_1.TIF]

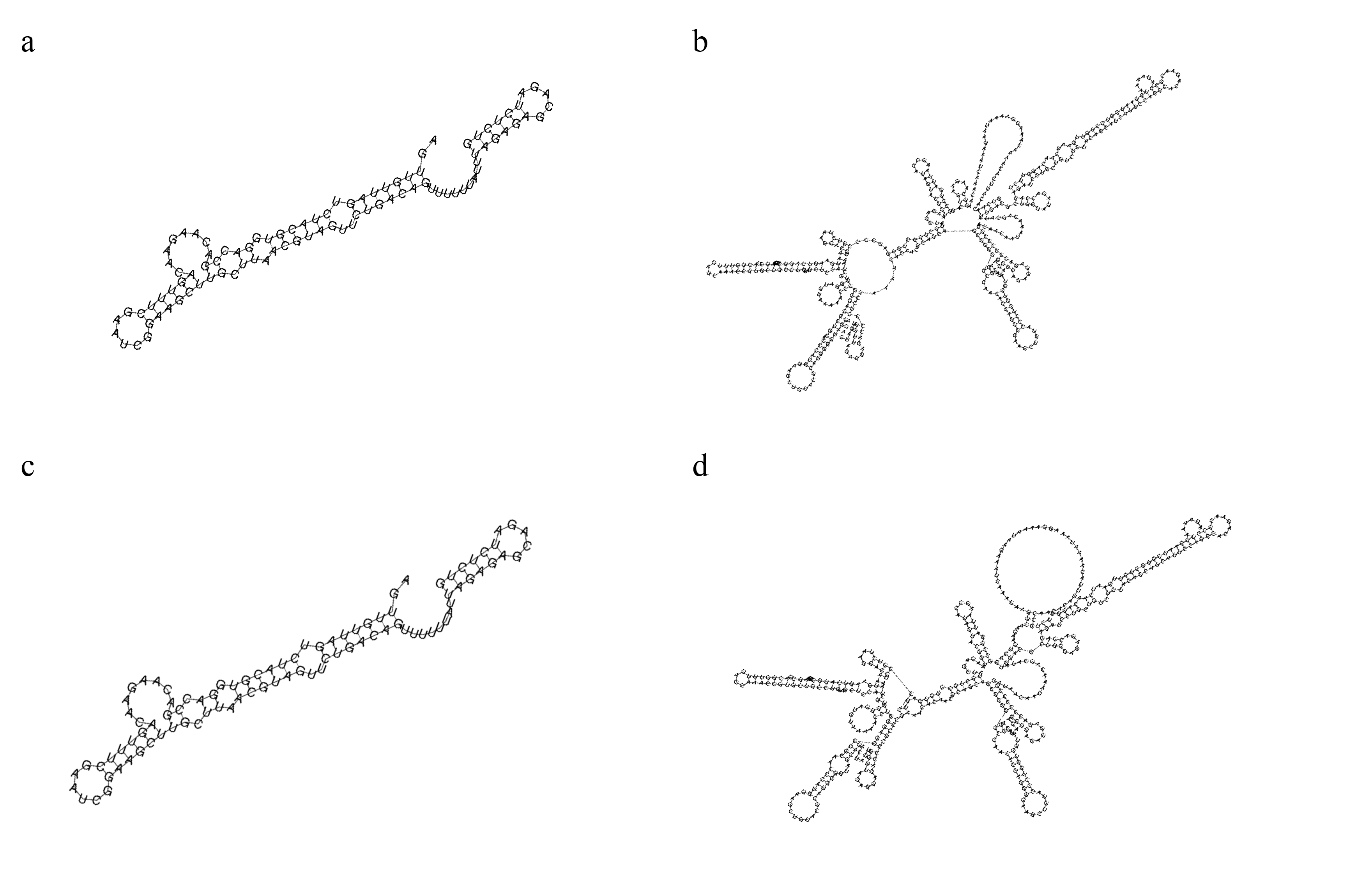

Supplement: Supplementary file 2 [file Image_2.TIF]

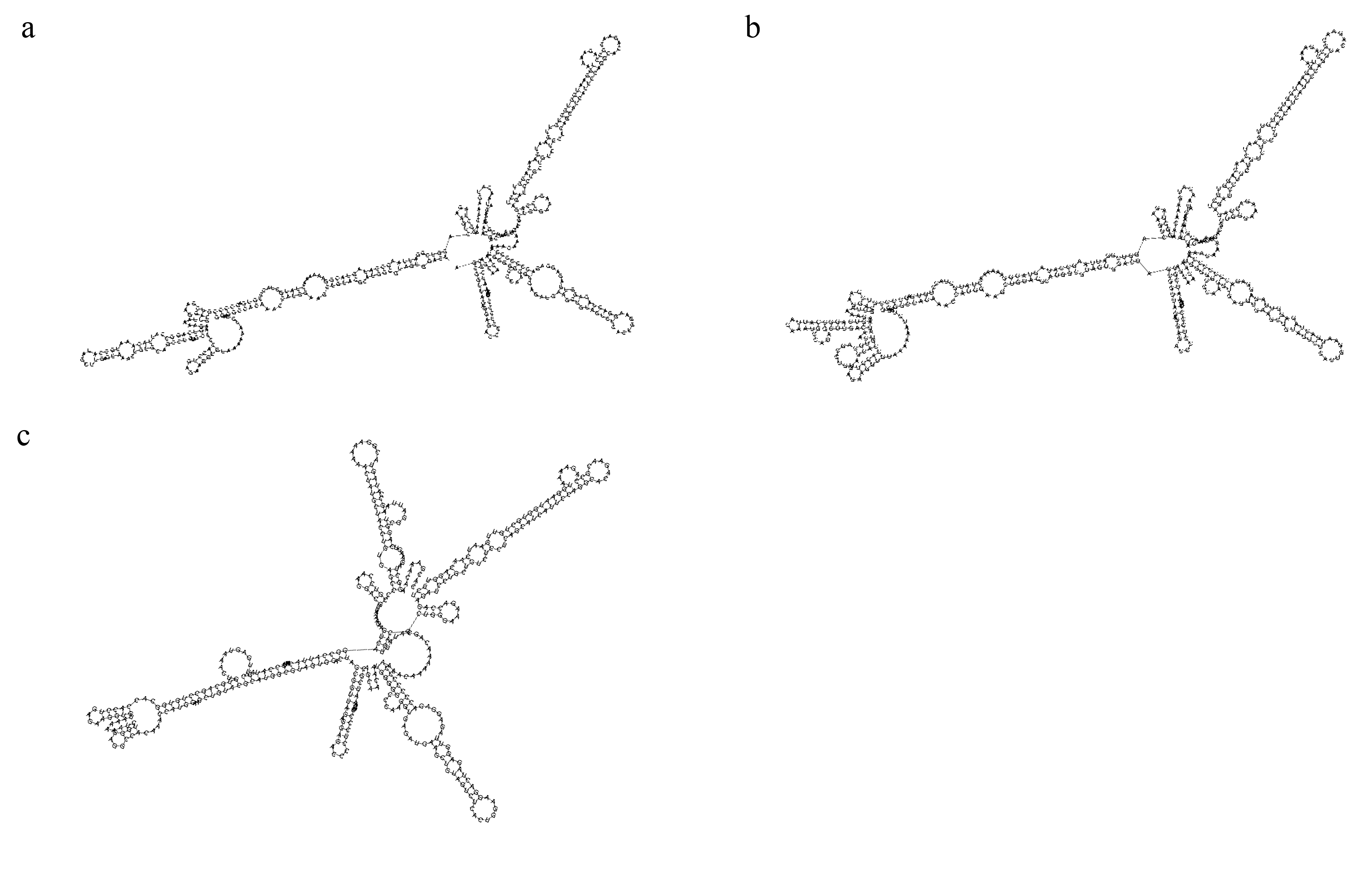

Supplement: Supplementary file 3 [file Image_3.TIF]

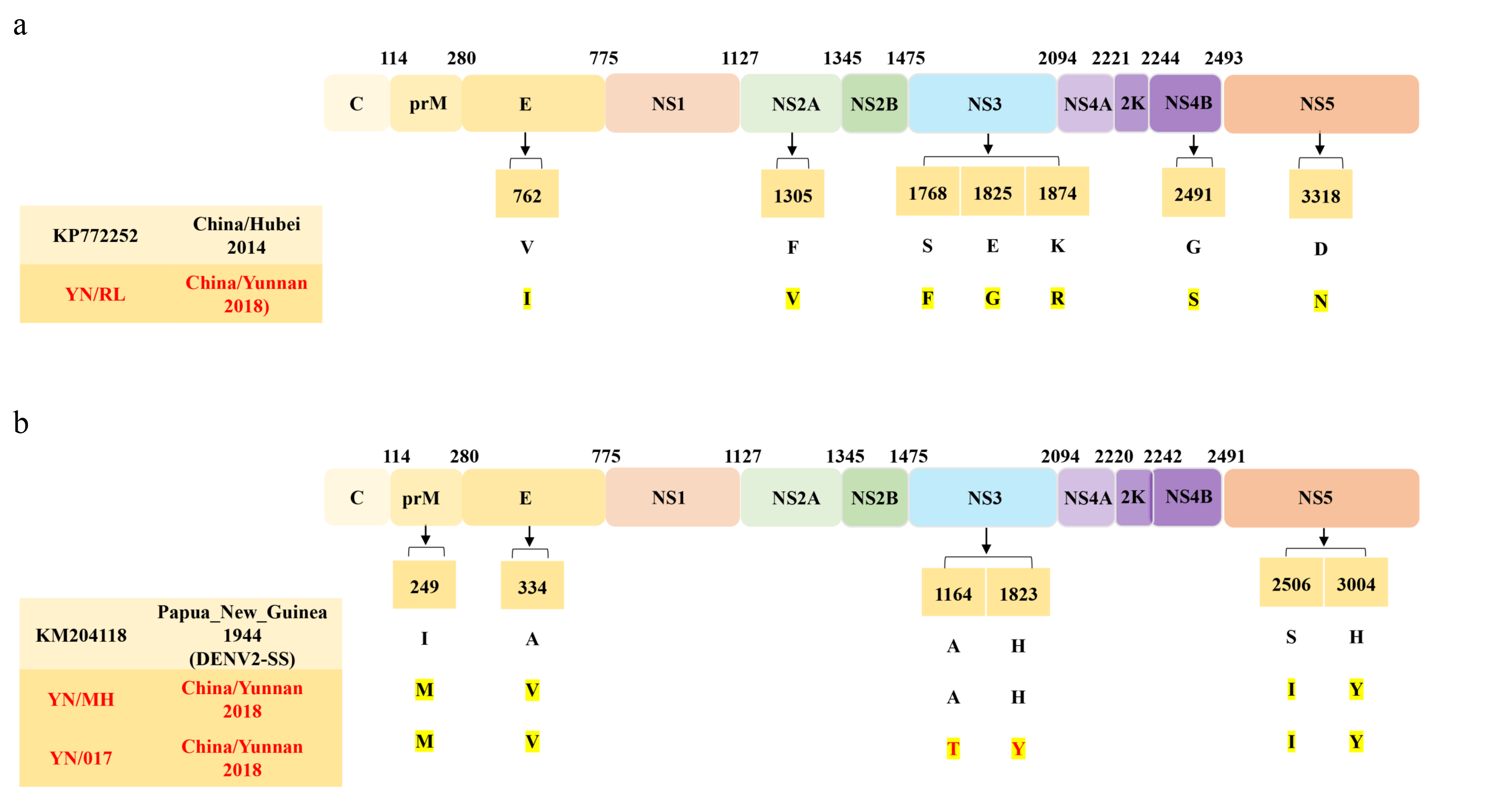

Supplement: Supplementary file 4 [file Image_4.TIF]
