# Supplementary material for: Retrospective investigation of the origin and epidemiology of the dengue outbreak in Yunnan, China from 2017 to 2018
Source: Front Vet Sci. 2023 Apr 3;10:1137392. doi: 10.3389/fvets.2023.1137392 (PMC10132138; doi:10.3389/fvets.2023.1137392)
Supplement: Supplementary file 5 [file Table_1.DOCX]

Table S1. List of primer sequences used in this study.

| Primer | Sequence (5’-3’) | Amplicon length (bp) |
| --- | --- | --- |
| DENV-JCF | TCAATATGCTGAAACGCGCGAGAAACCG | 511 |
| DENV-JCR | TTGCACCAACAGTCAATGTCTTCAGGTTC |  |
| DENV1-EF | TGCCATAGGAACATCCATCAC | 1600 |
| DENV1-ER | TCCCAATGGCTGCTGATAGTC |  |
| DENV2-EF | GACCTTGGTGARTTGTGTGAAG | 2055 |
| DENV2-ER | GACATTGATTATTGAC |  |
