# Supplementary material for: Retrospective investigation of the origin and epidemiology of the dengue outbreak in Yunnan, China from 2017 to 2018
Source: Front Vet Sci. 2023 Apr 3;10:1137392. doi: 10.3389/fvets.2023.1137392 (PMC10132138; doi:10.3389/fvets.2023.1137392)
Supplement: Supplementary file 6 [file Table_2.DOCX]

Table S2. Primer sequence for the amplification and sequencing of the DENV1 genome.

| Primer | Sequence (5’-3’) | Amplicon length (bp) |
| --- | --- | --- |
| DENV1-1F | AGTTGTTAGTCTACGTGGAC | 1500 |
| DENV1-1R | GTTCTAGGTGAACAATCCA |  |
| DENV1-2F | GTGTGTGACAAACTGGAA | 1430 |
| DENV1-2R | TTTTTTCCTTGAGCCAAGA |  |
| DENV1-3F | GAGGAGGGTGTGTGTGGAATTC | 1413 |
| DENV1-3R | AGCCATTGTCTTCCATGCA |  |
| DENV1-4F | GGACTTGCAATGGGCATTA | 1416 |
| DENV1-4R | CTTCACTGCTGTTGTTTGG |  |
| DENV1-5F | CAAGAAGATACCTTCCAGC | 1377 |
| DENV1-5R | AGGCTCTTCCTCCTTGTTC |  |
| DENV1-6F | CCCTGCGCGAGTTTAAAGA | 1487 |
| DENV1-6R | CTCTTCCACAACCGAGGTC |  |
| DENV1-7F | GGGATTATGGAAGTGGACA | 1428 |
| DENV1-7R | TTCCACTCCGCTGAGTGAA |  |
| DENV1-8F | GGAGCTTCACAAACAGGGA | 1103 |
| DENV1-8R | CTGTTGTCATCCATTGGTG |  |
| DENV1-9F | CTGGATGGAGCCTGAGAGA | 935 |
| DENV1-9R | AGAACCTGTTGATTCAACA |  |
