# Supplementary material for: Retrospective investigation of the origin and epidemiology of the dengue outbreak in Yunnan, China from 2017 to 2018
Source: Front Vet Sci. 2023 Apr 3;10:1137392. doi: 10.3389/fvets.2023.1137392 (PMC10132138; doi:10.3389/fvets.2023.1137392)
Supplement: Supplementary file 7 [file Table_3.DOCX]

Table S3. Primer sequence for the amplification and sequencing of the DENV2 genome.

| Primer | Sequence (5’-3’) | Amplicon length (bp) |
| --- | --- | --- |
| DENV2-1F | AGTTGTTAGTCTACGTGGACC | 1011 |
| DENV2-1R | RTAAGACTATGTCAACCCAGC |  |
| DENV2-2F | TTCAGGAGGAAGCTGGGTTG | 1450 |
| DENV2-2R | AACCACTATCGGCCTGCACC |  |
| DENV2-3F | TTGGGAGTTATGGTGCAGGC | 1085 |
| DENV2-3R | CCATGTCCGGCTGTGACCAA |  |
| DENV2-4F | GCCTCTGGAAAACTCATAACA | 1345 |
| DENV2-4R | CCACATTGTATGGAATGTTCC |  |
| DENV2-5F | CTGTGGGAAGTGAAGAAACAA | 1412 |
| DENV2-5R | CTTCCTATTCTCCCTCTTCTT |  |
| DENV2-6F | CTGACATTCAGAAATGGGTG | 1545 |
| DENV2-6R | GGTCAATCACTGTTATTCCAT |  |
| DENV2-7F | GCAACATCCTGGACATAGATC | 1370 |
| DENV2-7R | TTCACTGATGACACTATGTTC |  |
| DENV2-8F | GAAGCAGGACGAACACTCA | 1412 |
| DENV2-8R | CCTTCTCCCTCCATCTGTC |  |
| DENV2-9F | CCACATGGAAGGAGAACAC | 1490 |
| DENV2-9R | AGAACCTGTTGATTCAACA |  |
