# Supplementary material for: Retrospective investigation of the origin and epidemiology of the dengue outbreak in Yunnan, China from 2017 to 2018
Source: Front Vet Sci. 2023 Apr 3;10:1137392. doi: 10.3389/fvets.2023.1137392 (PMC10132138; doi:10.3389/fvets.2023.1137392)
Supplement: Supplementary file 8 [file Table_4.DOCX]

Table S4. Homologies of the complete ORF sequences of the YN/RL isolate.

| YN/RL2017 | ORF | C | prM | E | NS1 | NS2A | NS2B | NS3 | NS4A | NS4B | NS5 |
| --- | --- | --- | --- | --- | --- | --- | --- | --- | --- | --- | --- |
| DENV1  (Genotype I) | 97.2 | 98.2 | 97.4 | 97.5 | 97.3 | 96.7 | 96.7 | 96.8 | 96.3 | 97.5 | 97.4 |
| DENV1  (Genotype II) | 92.4 | 94.4 | 91.6 | 91.9 | 92.9 | 97 | 97.4 | 92.8 | 87.7 | 91 | 91.7 |
| DENV1  (Genotype III) | 92.3 | 93.8 | 93.8 | 91.8 | 91.9 | 90.6 | 92.8 | 93.2 | 91.6 | 92.4 | 92.2 |
| DENV1  (Genotype IV) | 91.3 | 94.1 | 91.6 | 90.6 | 92.4 | 89.4 | 90.8 | 91.7 | 89.5 | 90.6 | 92 |
| DENV1  (Genotype V) | 90.5 | 94.1 | 92 | 90.8 | 91.3 | 87.8 | 91.3 | 91.2 | 87.9 | 91 | 91.3 |
