# Supplementary material for: Retrospective investigation of the origin and epidemiology of the dengue outbreak in Yunnan, China from 2017 to 2018
Source: Front Vet Sci. 2023 Apr 3;10:1137392. doi: 10.3389/fvets.2023.1137392 (PMC10132138; doi:10.3389/fvets.2023.1137392)
Supplement: Supplementary file 9 [file Table_5.DOCX]

Table S5. Homologies of the complete ORF sequences of the YN/MH and YN/017 isolates.

| YN/MH and  YN/017 | ORF | C | prM | E | NS1 | NS2A | NS2B | NS3 | NS4A | NS4B | NS5 |
| --- | --- | --- | --- | --- | --- | --- | --- | --- | --- | --- | --- |
| DENV2  (America) | 91.8 | 94.1 | 92 | 91.9 | 92 | 91.4 | 89.5 | 91.6 | 91.3 | 91 | 92 |
| DENV2  (America/Asia) | 93.9 | 93.8 | 94.2 | 94.7 | 94.3 | 92.7 | 93.8 | 94 | 92.1 | 93.3 | 93.9 |
| DENV2  (Asia I) | 94.8 | 97.1 | 93.8 | 94.3 | 94.5 | 94.3 | 96.7 | 94.6 | 95.8 | 93.4 | 95.3 |
| DENV2  (Asia II) | 99.8 | 99.7 | 99.4 | 99.8 | 99.7 | 99.7 | 100 | 99.9 | 99.7 | 99.6 | 99.9 |
| DENV2  (Cosmopolitan) | 93.1 | 93.3 | 94.8 | 94.2 | 93.1 | 91.6 | 91.3 | 92.5 | 91.1 | 92.9 | 93.4 |
| DENV2  (Sylvatic) | 81.7 | 86.2 | 81.5 | 83.2 | 81.8 | 78 | 81.3 | 81.1 | 79 | 79.9 | 82.6 |
